# Estimation of treatment effect in presence of noncompliance and competing risks: a simulation study

**DOI:** 10.1038/s41598-023-40538-2

**Published:** 2023-08-18

**Authors:** Malihe Safari, Habib Esmaeili, Hossein Mahjub, Ghodratollah Roshanaei

**Affiliations:** 1 Department of Biostatistics, School of Medicine, Arak University of Medical Sciences, Arak, Iran; 2 Staburo GmbH, Munich, Germany; 3 Department of Biostatistics, Faculty of Public Health, Research Center for Health Sciences, Hamadan University of Medical Sciences, Hamadan, Iran; 4 Department of Biostatistics, School of Public Health, Modeling of Noncommunicable Diseases Research Center, Hamadan University of Medical Sciences, Hamadan, Iran

**Keywords:** Diseases, Medical research, Oncology, Risk factors

## Abstract

A randomized controlled trial is commonly designed to assess the treatment effect in survival studies, in which patients are randomly assigned to the standard or the experimental treatment group. Upon disease progression, patients who have been randomized to standard treatment are allowed to switch to the experimental treatment. Treatment switching in a randomized controlled trial refers to a situation in which patients switch from their randomized treatment to another treatment. Often, the switchis from the control group to the experimental treatment. In this case, the treatment effect estimate is adjusted using either convenient naive methods such as intention-to-treat, per-protocol or advanced methods such as rank preserving structural failure time (RPSFT) models. In previous simulation studies performed so far, there was only one possible outcome for patients. However, in oncology in particular, multiple outcomes are potentially possible. These outcomes are called competing risks. This aspect has not been considered in previous studies when determining the effect of a treatment in the presence of noncompliance. This study aimed to extend the RPSFT method using a two-dimensional G-estimation in the presence of competing risks. The RPSFT method was extended for two events, the event of interest and the competing event. For this purpose, the RPSFT method was applied based on the cause-specific hazard approach, the result of which is compared to the naive methods used in simulation studies. The results show that the proposed method has a good performance compared to other methods.

## Introduction

Randomized controlled trials (RCTs) are used to evaluate the effect of a new therapy and the efficacy and safety of experimental treatment versus control treatment. In survival analysis, there are three approaches, namely parametric, semi-parametric and nonparametric to analyze time-to-event outcomes. A semi-parametric model is a statistical model that contains both parametric and nonparametric components. A well-known example of a semi-parametric model is the Cox proportional hazards model^1,2^.

In RCTs, if all patients remain in their randomized allocated group until the end of the follow-up period and fully adhere to their assigned treatment, naive methods for estimating treatment effect, such as the log-rank test, may not be subject to bias. In reality, however, some patients may switch from their assigned treatment to the other treatment and/or discontinue treatment due to disease progression or other reasons. In most cases, switching from the standard treatment to the experimental treatment is decided by the investigator or the Ethics Committee^3^. In case of switching, the effect of treatment can be corrected using naive methods such as per-protocol (PP) or intention-to-treat (ITT) method and/or advanced methods such as rank preserving structural failure time (RPSFT) method. Each method treats the switched patients using a different procedure^4–7^. In the ITT method, patients are analyzed according to their randomized assigned treatment, regardless of whether they received that treatment.

The main advantage of this method is that the randomization is preserved, but it does not directly address the impact of switchers on the estimate of efficacy when comparing the experimental treatment and the standard treatment. This may lead to underestimate the appropriate efficacy of a new treatment^3,5,8^.

The first approach of PP analysis includes those patients who follow their assigned treatment and excludes completely patients who do not follow the treatment from the analysis (PP-ex approach). This approach may induce selection bias if switching is concerned with prognostic factors. In the second of approach of PP, patients who switched treatment groups are censored (PP-cen approach). Therefore, prognosis is likely different for patients who switch treatment, which is prone to bias^8,9^. Robins and Tsiatis developed the RPSFT method based on G-estimation (GE) to estimate the causal treatment effect in the presence of noncompliance^6^. Several simulation studies have been conducted to evaluate the performance of these methods in estimating the treatment effect in different scenarios and using different criteria^10–16^. Odondi et al. (2010) investigated different methods to correct treatment effect with noncompliance and showed that the RPSFT method had less bias and a higher percentage of coverage compared to other methods^17^. Morden et al. (2011) showed in a simulation study that the naive methods are often inappropriate when dealing with treatment switching, and thus proposed alternative approaches such as the iterative parameter estimation (IPE) and the RPSFT method to adjust the estimates^3^. Wu et al. (2014) evaluated the treatment effect in a three-arm clinical trial in the presence of noncompliance. They studied the ITT, PP and RPSFT methods and indicated that the RPSFT method performed well, while ITT and PP methods did not guarantee reliable results^18^. Zhang et al. (2016) performed a simulation study to compare different methods. They proposed modified IPE (MIPE) and showed that the performance of MIPE, IPE and RPSFT was better than that of naive methods^19^. Latimer et al. (2017) compared the performance of the PP, ITT, RPSFT, IPE, structural nested model (SNM), inverse probability of censoring weights (IPCW) and the two-stage estimation (TSE) method in a simulation study. They showed that the RPSFT and IPE generate low bias when the treatment effect is time-independent. In a scenario with a high proportion of switchers, the IPCW and SNM exhibited a high bias. They also showed that TSE generated low bias in all scenarios, which was an appropriate adjustment method^12^. Latimer et al. (2018) in comprehensive simulation scenarios, concluded that RPSFT, IPCW, and TSE generated lower bias than the ITT. They also showed that the estimation of treatment effect by Accelerated Failure Time (AFT) -based models had higher accuracy than Hazard Ratio-based models^13^. Latimer et al. (2019) examined the performance of the RPSFT and TSE with and without re-censoring in a simulation study. They showed that the analyses with re-censoring had a negative bias, while the analyses in lack of re-censoring generated a positive bias. They concluded that the analyses should be performed with and without re-censoring^14^. Latimer et al. (2020) introduced a new version of TSE, using SNM and G-estimation. They compared this method with the simple TSE, RPSFT, and IPCW method. They concluded that in scenarios with time-dependent confounding variables, the TSE and RPSFT had substantial bias, while in scenarios with no time-dependent confounding variables, all methods performed well^15^.

In classical time-to-event or survival analyses, subjects are at risk of a terminal event. In this case, the usual classical techniques such as Kaplan–Meier, log-rank test, and Cox proportional hazards model are used to analyze survival data. However, in many oncology trials some patients are at risk for more than one mutually exclusive event or endpoint. For example, time to a cancer-specific death is considered as primary endpoint. A competing event is an event whose occurrence either precludes the occurrence of another event under examination or fundamentally alters the probability of this other event. However, other events—so-called competing risks (CR)—may preclude the occurrence of the event of interest^1,2^.

Current techniques ignore the event type, and combine multiple primary endpoints into a single composite endpoint. Composite endpoints are defined as experiencing any events among a given set of competing events in a follow-up period. In the presence of CR, the use of classical analysis based on composite endpoints leads to bias. If the competing events are heterogeneous, that means, endpoints have different importance for patients or they show different treatment effects, there will be a risk of misinterpretation of results^20^.

CR analyses allow extracting the contribution of a treatment on each competing event type separately. In the context of CR, the two common methods of the cause-specific hazard (CSH) regression model and the sub-distribution hazard (SDH) regression model are often used^1,2^. So far, in all previous simulation studies with noncompliance conducted to assess the treatment effect, only one cause of event has been considered until the end of study period. The estimation of treatment effect with adjustment methods in the presence of noncompliance and competing risks is problematic and cannot be recommended due to the competing events being ignored. Hence, the mentioned methods for noncompliance should be accounted for the CR.

Among the various proposed methods in noncompliance simulation studies, the RPSFT method and the GE algorithm appear to have gained popularity and produced the best results in situations where the treatment effect was time-independent^11^. It is unclear how the RPSFT method performs in the presence of CRs. In this paper, both the RPSFT and naïve methods were evaluated using a simulation study in the presence of competing events. That is, the RPSFT method was extended based on the GE algorithm for RCTs in the presence of CRs.

The standard approach to assess the impact of prognostic variables on CR is to assume that each CSH follows a proportional hazards model. Assuming proportional CSH models, the proportional SDH models are misspecified. In this article, to study competing risks, data were generated based on CSH with the noncompliance assumption and with respect to disease progression in RCTs^21–25^. Hence, this study extends the RPSFT method based on CSH models for CRs.

This article is organized as follows: in Section “Notation and proposed method” a method is presented to estimate the treatment effect when there are noncompliance and competing events; the setting of the simulation study is discussed in Section “Simulation study design”; results of simulation study are described in Section “Simulation study results”; discussion on results and conclusion of this study is given in Section “Case Study: a randomized clinical trial in colorectal cancer”.

## Notation and proposed method

### Notation

Suppose a clinical trial was conducted to evaluate the efficacy of a treatment on survival of patients in standard and experimental treatment groups. After randomization, each patient in the standard treatment could switch to the experimental treatment at time t (t > 0). If ${\mathrm{G}}_{\mathrm{i}}$ denotes the randomized assigned treatment group for patient $i$(${\mathrm{G}}_{\mathrm{i}}=1$ for experimental treatment and ${\mathrm{G}}_{\mathrm{i}}=0$ for standard treatment), actual treatment indicator for subject $i$ at time t can be defined as follows:

1 $${\mathrm{A}}_{\mathrm{i}}\left(\mathrm{t}\right)=\left\{\begin{array}{c}1 \; for\; patient \; i \; received \; exprimental \; treatment \; at \; time \; t\\ 0 \; for \; patient \; i \; received \; standard \; treatment \; at \; time \; t\end{array}\right.$$

So that, $${\mathrm{A}}_{\mathrm{i}}\left(0\right)={\mathrm{ G}}_{\mathrm{i}}$$.

Here, $i\in \{1, ...,n\}$ indices the n study subject entered in the trial and followed-up for a period of time. It was assumed that attaining the end of study without any failure events was the only source of censoring (administrative censoring). Each patient was followed-up from randomization to experience the events or end of study, whichever occurred first.

Also each patient may experience the event of interest, a competing event or is censored at the end of study without any event. The time interval between the entrance time point and the end of study is represented by ${C}_{i}$; and corresponding survival time is ${\mathrm{X}}_{\mathrm{i}}$. The observed survival time is ${\mathrm{T}}_{\mathrm{i}}{=\mathrm{min}( {\mathrm{X}}_{\mathrm{i}},\mathrm{C}}_{\mathrm{i}})$ with ${\updelta }_{\mathrm{i}}=1$ if the event of interest is observed, ${\updelta }_{\mathrm{i}}=2$ when the competing event is observed and ${\updelta }_{\mathrm{i}}=0$ otherwise (i.e. when a failure event occurs after the end of study). Then,

2 $${\updelta }_{\mathrm{i}}=\left\{\begin{array}{cc}1& if \; the \; event \; of \; interest \; is \; observed\; (cause\; 1) \\ 2& if \; the \; competing \; event \; is \; observed \; (cause\; 2) \\ 0& if \; the \; survival \; time \; is \; censored\end{array}\right.$$

Thus, the observed data for subject *i* are $\left\{{\mathrm{G}}_{\mathrm{i}},{\mathrm{A}}_{\mathrm{i}}\left(\mathrm{t}\right),{\mathrm{T}}_{\mathrm{i}},{\updelta }_{\mathrm{i}}\right\}$. Suppose that the experimental treatment has a multiplicative effect on the survival time, which as in the accelerated failure time models, is represented by ${e}^{\psi }$. Assume that the distribution function of the survival time in standard treatment group is ${F}_{\theta }(t)$ , where the parameter $\uptheta$ is unknown. So the distribution function of survival time and corresponding density function in the experimental treatment group will be ${F}_{\uptheta }\left({e}^{\psi }\mathrm{t}\right)$ and ${f}_{\uptheta }\left({e}^{\psi }\mathrm{t}\right)$, respectively. Therefore, the likelihood for the ${i}^{th}$ patient depending on being in either the standard or the experimental treatment in classical survival analysis is defined as below:

3 $$Pr\left(t,\updelta \right)={\left[{f}_{\uptheta }(\mathrm{t})\right]}^{{\updelta }_{\mathrm{i}}}\times {[1-{F}_{\uptheta }(\mathrm{t})]}^{1-{\updelta }_{\mathrm{i}}}$$

Or

4 $$Pr\left( {t,\delta } \right) = \left[ {e^{\psi } f_{\theta } \left( {e^{\psi } t} \right)} \right]^{{\delta_{i} }} \times \left[ {1 - F_{\theta } \left( {e^{\psi } t} \right)} \right]^{{1 - \delta_{i} }}$$

On the other hand, some patients may be switched from their randomized assigned treatment to the other treatment, following the diagnosis and decision of the clinical physician. The switching usually occurs from the standard treatment to the experimental treatment group^6^. We consider a situation where a patient with survival time T_i_ switches from the standard to the experimental treatment at time W_i_. The latent survival time is adjusted by the treatment effect ${\mathrm{e}}^{-\uppsi }$ as follows:

5 $${U}_{\mathrm{i}}={\mathrm{W}}_{\mathrm{i}}+{e}^{\psi }({T}_{\mathrm{i}}-{\mathrm{W}}_{\mathrm{i}})$$

Thus, the observed survival time will be resulted as:

6 $${T}_{i}={W}_{i}+{e}^{-\psi }\left({U}_{i}-{\mathrm{W}}_{i}\right)$$

The random variable ${U}_{\mathrm{i}}$ (i.e. latent survival time), is the lifetime of the ${i}^{th}$ patient if he/she never received treatment.

And so, the distribution of survival time for switched patient in the experimental treatment will be as follows:

7 $$\begin{aligned} Pr\left( {T_{i} \le t} \right) = \; & Pr \; \left( {W_{i} + e^{ - \psi } \left( {U_{i} - W_{i} } \right) \le t} \right) \\ = \; & Pr \; \left( {U_{i} \le W_{i} + e^{\psi } \left( {t - W_{i} } \right)} \right) = F_{\theta } \; \left( {e^{\psi } \left[ {W_{i} + e^{\psi } \left( {t - W_{i} } \right)} \right]} \right) \\ = \; & F_{\theta } \left( {e^{\psi } W_{i} + e^{2\psi } \left( {t - W_{i} } \right)]} \right) \\ \end{aligned}$$

So, if the treatment is beneficial, the survival time for a switched patient to the experimental treatment will be increased. Thus the likelihood for the ${i}^{th}$ switched patient in experimental treatment in classical survival analysis will be as following:

8 $$Pr\left( {t,\delta_{i} ,\theta , \psi } \right) = \left[ {e^{2\psi } f_{\theta } \left( {e^{\psi } W_{i} + e^{2\psi } \left( {t_{i} - W_{i} } \right)} \right)} \right]^{{\delta_{i} }} \times \left[ {1 - F_{\theta } \left( {e^{\psi } W_{i} + e^{2\psi } \left( {t_{i} - W_{i} } \right)} \right)} \right]^{{1 - \delta_{i} }}$$

On the other hand, the likelihood function for n patients in ${j}^{th}$ competing risk ($j=1, ...,k$) is

9 $$L=\prod_{i=1}^{n}\left(\left(\prod_{j=1}^{k}{{f}_{\theta }(t)}^{{I(\delta }_{i}=j)}\right){(1-{F}_{\theta }(t))}^{1-{I(\delta }_{i}=j)}\right)$$

Accordingly, the contribution of the ${i}^{th}$ patient to the likelihood in presence of both competing risk and noncompliance can be written as follows:

$$\mathcal{L}=\prod_{i=1}^{n}Pr\left({t}_{i},{\delta }_{i}\right)=\prod_{i=1}^{n}{\left[{f}_{\theta }(t)\right]}^{{\delta }_{i}}{[1-{F}_{\theta }(t)]}^{1-{\delta }_{i}}$$

10 $$=\left\{\prod_{\begin{array}{c}i:{W}_{i}=\infty \\ {G}_{i}=\mathrm{0,1}\end{array}}^{n}\left[\prod_{j=1}^{k}{\left[{e}^{{\psi }_{j}{G}_{i}}{f}_{{\theta }_{j}}\left({e}^{{\psi }_{j}{G}_{i}} t\right)\right]}^{{I(\delta }_{i}=j)}{\left[1-{F}_{{\theta }_{j}}\left({e}^{{\psi }_{j}{G}_{i}} t\right)\right]}^{1-{I(\delta }_{i}=j)}\right]\right\} \times \left\{\prod_{\begin{array}{c}i:{W}_{i}<\infty \end{array}}{\left[{e}^{2{\psi }_{j}}{f}_{{\theta }_{j}}({e}^{{\psi }_{j}}{W}_{i}+{e}^{2{\psi }_{j}}({t}_{i}-{W}_{i}))\right]}^{{I(\delta }_{i}=j)}{\left[1-{F}_{{\theta }_{j}}({e}^{{\psi }_{j}}{W}_{i}+{e}^{2{\psi }_{j}}({t}_{i}-{W}_{i}))\right]}^{1-{I(\delta }_{i}=j)}\right\}$$

Aforementioned likelihood is the product of two terms, the first term is for patients that never switched and the second term is for the switched patients, where $i\in \{1, ...,n\}$ shows the index for patients entered into the trial and $j\in \{1, ...,k\}$ shows the index for number of competing events. In this paper, k = 2 is assumed. Also ${e}^{{\psi }_{j}}$ is an unknown parameter representing the effect of the experimental treatment versus the standard treatment in ${j}^{th}$ competing event.

If ${\psi }^{T}=\left({\psi }_{1},{\psi }_{2},\cdots ,{\psi }_{k}\right) \mathrm{and} \lambda =\left(\theta ,{\psi }^{T}\right)$ were parameters vector, then to estimate of parameters following equation should be solved:

11 $$\frac{\partial \; \mathit{log} \; \mathcal{L}(\lambda )}{\partial \lambda }=0$$

Under some regularity conditions $,$ the maximum likelihood estimator $\widehat{\lambda }$ is consistent and asymptotically normal with the mean $\uplambda$ and variance covariance matrix:

12 $$\left[ {E\frac{{\partial^{2} \log {\mathcal{L}}\left( \lambda \right)}}{{\partial \lambda \; \partial \lambda^{,} }}} \right]^{ - 1} Var \left[ {\frac{{\partial \log {\mathcal{L}}\left( \lambda \right)}}{\partial \lambda }} \right]\left[ {E\frac{{\partial^{2} \log {\mathcal{L}}\left( \lambda \right)}}{{\partial \lambda \partial \lambda^{,} }}} \right]^{ - 1}$$

One approach for solving the likelihood equation in (12) is GE algorithm in structural models, which is presented in next section.

### The Proposed Method: Extension of rank preserving structural failure times models (RPSFT) to two-dimensional

Robins and Tsiatis (1991) developed the RPSFT method to estimate the causal treatment effect in the presence of noncompliance. Their method considers only a single cause of failure and is used when patients discontinue treatment according to the protocol but receive a treatment which they are not allocated to^6^. An appropriate CR method is alternatively applied, when multiple causes of failure exist.

In this paper, RPSFT method was applied based on CSH approach and adjusted in the presence of CR. This means, the RPSFT method was extended for two different events, the event of interest and the competing event.

In RPSFT, random variable ${\mathrm{U}}_{\mathrm{i}}$ is the lifetime of the ${i}^{th}$ patient if he/she had never been received any treatments (i.e. ${A}_{i}\left(t\right)=0$ for all t > 0). Indeed, it is assumed that the latent survival time ${\mathrm{U}}_{\mathrm{i}}$ does not depend on the treatment assignment. In an ideal double-blind randomized clinical trial, it is hoped that this assumption hold true; because the counterfactual latent time ${\mathrm{U}}_{\mathrm{i}}$ is a fixed characteristic of the patient *i*, and it is unaffected by the treatment group or the actual treatment history. Hence for a patient in the experimental treatment with observed survival time ${\mathrm{T}}_{\mathrm{i}},$ the latent time ${U}_{i}={e}^{\psi }{T}_{i}$ and in general form can be written as follows:

13 $$U_{i} = \mathop \int \limits_{0}^{{T_{i} }} exp\left[ {\psi A_{i} \left( t \right)} \right]dt$$

Here, $A_{i} \left( t \right)$ (see function 1) is the factual treatment status at time t, and $\psi$ ($- \infty < \psi < + \infty$) is an unknown parameter representing the causal effect of the experimental versus the standard treatment^6^.

To extend the RPSFT model and GE algorithm for CR, ${\text{U}}_{{\text{i}}}$ is rewritten as follows:

14 $$U_{i} = \mathop \int \limits_{0}^{{T_{i} }} exp\left[ {\psi^{T} A_{i} \left( t \right)p_{i} } \right]dt$$

Here,${\psi }^{T}=({\psi }_{E},{\psi }_{C})$ is the transpose vector of the unknown parameters that represents the impact of treatment on failure time of the event of interest (E) and the competing event (C), respectively. ${A}_{i}\left(t\right)$ is a treatment indicator and ${\mathrm{p}}_{\mathrm{i}}$ determines the type of CR defined as follows:

15 $${p}_{i}=\left\{\begin{array}{cc}(\mathrm{1,0})& if \; the \; event \; of \;interest \; occurs\\ (\mathrm{0,1})& if \; the \; competing \; event \; occurs\end{array}\right.$$

Namely, when ${A}_{i}\left(t\right)=0$ for all t > 0, ${T}_{i}={U}_{i}$. When ${\mathrm{A}}_{\mathrm{i}}\left(\mathrm{t}\right)=1$ for all t > 0 and the event of interest occurs (i.e. ${p}_{i}=\left(\mathrm{1,0}\right)$), then, ${T}_{i}={U}_{i}\mathit{exp}\left({-\psi }_{E}\right)$, but when a competing event occurs (i.e. ${p}_{i}=\left(\mathrm{0,1}\right)$), then ${T}_{i}={U}_{i}\mathit{exp}\left({-\psi }_{C}\right)$. That means, $\mathit{exp}\left({-\psi }_{E}\right)$ is the multiplicative factor increasing or decreasing failure time for the event of interest and $\mathit{exp}\left({-\psi }_{C}\right)$ is the multiplicative factor increasing or decreasing failure time for the competing event when comparing experimental versus standard treatment. Here, ${\psi }_{k}=0$ indicates that the treatment is neither beneficial nor detrimental; ${\psi }_{k}<0$ indicates that the experimental treatment is beneficial and increases a subject’s time of failure for event type k. If ${\psi }_{k}>0$, the experimental treatment is detrimental and decreases patients’ time of failure for event type k. It should be noted that when the underlying distribution of ${U}_{i}$ is an exponential distribution, ${\psi }_{E}$ is equal to the log-hazard ratio of the hazard function of experimental to standard treatment for the event of interest ${(\psi }_{E}=\mathit{log}\left({h}_{m1}/{h}_{s1}\right)$ and ${\uppsi }_{C}$ is equal to the log-hazard ratio of the hazard function of experimental to standard treatment for the competing event ${(\psi }_{C}=\mathit{log}\left({h}_{m2}/{h}_{s2}\right)$, where ${h}_{m1}$ and ${h}_{s1}$ are hazard rate of the event of the interest for the experimental and standard treatment, respectively. Also,${h}_{m2}$ and ${h}_{s2}$ are hazard rates of the competing event in experimental and standard treatment, respectively.

The ${\psi }_{E}$ and ${\psi }_{C}$ are estimated under the assumption in which the distribution of the latent survival time ${U}_{i}$ is independent of the assigned treatment group ${G}_{i}$^11,27^ (see function 16)

16 $$Pr\left( {U_{i} \ge t{|}G_{i} = 0} \right) = Pr\left( {U_{i} \ge t{|}G_{i} = 1} \right) , \; for \; all \; t \ge 0$$

In other words, for a given parameter $({\psi }_{E},{\psi }_{C}),$ the random variable ${\mathrm{U}}_{\mathrm{i}}({\psi }_{E},{\psi }_{C})$ is independent from ${\mathrm{G}}_{\mathrm{i}}$ (i.e. the counterfactual latent time of patients is independent from the treatment group to which they were randomized). Thus, estimation of $({\psi }_{E},{\psi }_{C})$ can be computed to test the equality of the distribution of the ${U}_{i}({\psi }_{E},{\psi }_{C})$ in the two groups using proportional hazards model:

17 $${h}_{i}\left(t=u\right)={h}_{0}(t){e}^{\left({\psi }_{E},{\psi }_{C}\right) {A}_{i}\left(t\right){ e}_{i}}$$

where ${U}_{i}({\psi }_{E},{\psi }_{C})$ is calculated for a grid of possible values $({\psi }_{E},{\psi }_{C})$, then ${U}_{i}({\psi }_{E},{\psi }_{C})$ are compared in the two treatment groups for a given parameter constellation $({\psi }_{j},{\psi }_{l})$. ${U}_{i}({\psi }_{j},{\psi }_{l})$ is computed as follows:

$${U}_{i}\left({\psi }_{j},{\psi }_{l}\right)=$$

18 $$\left\{ {\begin{array}{*{20}c} {exp\left( {\psi _{j} } \right) \times T_{i}^{{exposed}} } & {amp;for\;experimental\;treatment\;patients\;with\;the\;event\;of\;interest} \\ {exp\left( {\psi _{l} } \right) \times T_{i}^{{exposed}} } & {for\;experimental\;treatment\;patients\;with\;competing\;event} \\ {T_{i}^{{unexposed}} } & {for\;standard\;treatment\;patients\;who\;never\;switched} \\ {exp\left( {\psi _{j} } \right) \times T_{i}^{{exposed}} + T_{i}^{{unexposed}} } & {for\;standard\;treatment\;patients\;who\;switched\;with\;the\;event\;of\;interest} \\ {exp\left( {\psi _{l} } \right) \times T_{i}^{{exposed}} + T_{i}^{{unexposed}} } & {for\;standard\;treatment\;patients\;who\;switched\;with\;competing\;event} \\ \end{array} } \right.$$

where: ${T}_{i}^{exposed}:$ Time spent in the experimental treatment. ${T}_{i}^{unexposed}:$ Time spent in the standard treatment.

The estimations for ${\psi }_{E}$ and ${\psi }_{C}$ are the values of $({\psi }_{j},{\psi }_{k})$, in which the p-value of the test is maximized and can be found by a search over values of a grid^28,29^. This search for values of $({\psi }_{j},{\psi }_{k})$ is continued until all possible values have been tested. A grid search is conducted over many pre-specified values of $({\psi }_{j},{\psi }_{k})$ (e.g., both are varying from -3 to 3 by increments of 0.001). This estimation procedure is called a two-dimensional G-estimation (GE)^30^.

As in the one-dimensional RPSFT, a new censoring time in two-dimensional ${C}_{i}({\psi }_{E},{\psi }_{C})={C}_{i}$ is defined when $min({\psi }_{E},{\psi }_{C})>0$, ${C}_{i}\left({\psi }_{E}\right)={C}_{i}\mathit{exp}\left({\psi }_{E}\right)$ when ${\psi }_{E}<{\psi }_{C}<0$ or ${\psi }_{E}<0<{\psi }_{C}$ for interest event, and ${C}_{i}\left({\psi }_{C}\right)={C}_{i} exp({\psi }_{C})$ when ${\psi }_{C}<{\psi }_{E}<0$ or ${\psi }_{C}<0<{\psi }_{E}$ for competing event. So the censoring time can be summarized as follows:

19 $${C}_{i}\left({\psi }_{E},{\psi }_{C}\right)= \left\{\begin{array}{c}{C}_{i} \; if \; min \; \left({\psi }_{E},{\psi }_{C}\right)>0 \\ {C}_{i}\times \mathit{exp}\left({\psi }_{E}\right) \; if \; {\psi }_{E}<{\psi }_{C}<0 \; or \; {\psi }_{E}<0<{\psi }_{C} \\ {C}_{i}\times \mathit{exp}\left({\psi }_{C}\right) \; if \; {\psi }_{C}<{\psi }_{E}<0 \; or \; {\psi }_{C}<0<{\psi }_{E}\end{array}\right.$$

Therefore, the adjusted observed survival time for ${i}^{th}$ patient can be written as:

20 $$X_{i} \left( {\psi_{E} ,\psi_{C} } \right) = \left\{ {\begin{array}{*{20}c} {\begin{array}{*{20}c} {\min \left[ {U_{i} \left( {\psi_{E} ,\psi_{C} } \right),C_{i} \left( {\psi_{E} } \right)} \right]} & {for \; interest \; event} \\ {\min \left[ {U_{i} \left( {\psi_{E} ,\psi_{C} } \right),C_{i} \left( {\psi_{C} } \right)} \right]} & {for \; competing \; event} \\ {\min\left[ {C_{i} \left( {\psi_{E} } \right),C_{i} \left( {\psi_{C} } \right)} \right]} & {for \; censored} \\ \end{array} } \\ \end{array} } \right.$$

Based on adjusting for switching from standard to experimental treatment, the adjusted variable of censoring is expressed as follows:

21 $${\Delta }_{i}\left({\psi }_{E},{\psi }_{C}\right)=\left\{\begin{array}{c}\begin{array}{cc}1& if \; {X}_{i}\left({\psi }_{E},{\psi }_{C}\right)={U}_{i}\left({\psi }_{E},{\psi }_{C}\right) \; for \; interest \; event\\ 2& if {X}_{i}\left({\psi }_{E},{\psi }_{C}\right)={U}_{i}\left({\psi }_{E},{\psi }_{C}\right) \; for \; competing \; event\\ 0& otherwise\end{array}\end{array}\right.$$

For patients who experienced the event of interest, ${\Delta }_{i}\left({\psi }_{E},{\psi }_{C}\right)=1$ and for the competing event ${\Delta }_{i}\left({\psi }_{E},{\psi }_{C}\right)=2$. We may have ${\Delta }_{i}\left({\psi }_{E},{\psi }_{C}\right)=0$ for non-zero values of ${\psi }_{E}$ and ${\psi }_{C}$; and patients who experienced an event. These patients will be censored artificially, which is called “re-censoring”.

For calculating the hazard ratio (HR), the observed survival time (${T}_{i}$) and the censoring variable (${\Delta }_{i}$) are used in the experimental treatment group. The adjusted observed survival time (${X}_{i}\left({\psi }_{E},{\psi }_{C}\right)$) and adjusted censoring variable (${\Delta }_{i}\left({\uppsi }_{E},{\uppsi }_{C}\right)$) are used for each patient in standard treatment group. Hence, re-censoring is used for each patient in the standard group^6^.

### Ethical approval

This study was approved by the Ethics Committee of Hamadan University of Medical Sciences (approval code: IR.UMSHA.REC.1398.637). All methods were performed in accordance with the relevant guidelines and regulations.

## Simulation study design

In order to evaluate the performance of the proposed method based on CSH in estimating treatment effect, the simulation study was designed with different scenarios. This was to compare the results of the proposed method with naïve methods (ITT and PP) in the presence of competing event. To understand the verification of the proposed method in CR, the event type was ignored but different causes of event were combined into a single event or composite endpoint, so called ITT-com, PPcen-com and RPSFT-com. The switching was only allowed from the standard to the experimental treatment. In PP method, all switched patients were considered censored at the time of switching (i.e. PP-cen method). Then, the performance of adjusting methods were assessed by criteria such as censoring percentage, coverage percentage, relative bias percentage and mean square error. The setting of this simulation study mimics a real cancer clinical trial in the presence of CR. All data management and analytical procedures were performed in the statistical programing software R version 3.5.3. Details are given below.

### Generation of survival time

Study data were simulated using the cause-specific hazards method according to the procedure presented by Beyersmann^24^. Constant hazards, hazard of patients with the event of interest (${h}_{m1},{h}_{s1}$) and hazard of patients with the competing event (${h}_{m2}$, ${h}_{s2}$) in experimental and standard treatment, were assumed respectively.

The generation of the survival time ${T}_{i}$ listed below:

1. The ${j}^{th}$ CSH i.e.${h}_{zj},\mathrm{for} z=m, s \mathrm{and } \; j=1, 2$ for the two treatment groups was considered.
2. The survival time T for each group was generated with all cause hazard ${h}_{z1}+{h}_{z2}$.
3. After generating the time, a binomial experiment was performed to decide the failure of the event of interest with the probability of ${h}_{z1}/{(h}_{z1}+{h}_{z2})$ in ${k}^{th}$ treatment group^24,26^.

The CSHs were considered such that they could generate approximately 50% of the event of interest and 20% of the competing event. 1000 data sets with 500 patients were simulated and randomized to either experimental or standard treatment groups with a 1:1 allocation scheme, to see if the methods provide accurate results on average. For each patient, entrance time was generated from a uniform distribution between 0 and 20 months and the end of study was considered 30 or 90 months. Only administrative censoring was considered in this study.

### Switching mechanism

It is assumed that patients could switch to experimental treatment in accordance with the protocol. The switching percentage were considered as 30%, 60% and 90%. The occurrence of switching for each patient in the standard treatment depended on the time of disease progression, which was generated as a proportion of survival times dictated by a random value between 0 and 1 taken from a beta distribution with alpha and beta parameters. The primary reason for generating disease progression times was to allow us to simulate switching time to be immediately after disease progression.

After generating all of the variables mentioned above, the survival time for the switched patients in the standard treatment should be adjusted. If time spent in the standard treatment is denoted by T^unexposed^ and the time spent in the experimental treatment is denoted by T^exposed^, then the total lifetime of the switched patient in the study should be calculated as follows:

$$T={T}^{\mathrm{unexposed}}+{e}^{-\psi }\times {T}^{\mathrm{exposed}}$$

where, ${e}^{-\psi }$ is the effect of experimental treatment. If a patient experiences the event of interest, then $\psi ={\psi }_{E}$, and if he/she experiences the competing event, $\psi ={\psi }_{C}$.

The HR can be converted to the treatment effect by using the formula in Collett, i.e. $\psi =\frac{ln(HR)}{\gamma }$ where γ is shape parameter and $\mathrm{exp}(-{\varvec{\psi}})$ is the treatment effect^31^.

Now, if the survival time is before the end of study, the patient will experience an event and the indicator variable of censoring takes the value k (k = 1, 2); it takes 0 and the patient is censored, otherwise.

### Considered scenarios in this study

Three scenarios were considered with different treatment effects for the event of interest and the competing event:

$${e}^{{-\uppsi }_{E}}=2 { (HR}_{E}=0.5) , {e}^{{-\uppsi }_{C}}=1 ({HR}_{C}=1)$$

$${e}^{{-\uppsi }_{E}}=2 { (HR}_{E}=0.5) , {e}^{-{\uppsi }_{C}}=0.5 ({HR}_{C}=2)$$

$$e^{{ - {\uppsi }_{E} }} = 2 (HR_{E} = 0.5) , e^{{ - {\uppsi }_{C} }} = 1.5\;\left( {HR_{C} = 0.6} \right)$$

So, 18 different scenarios were simulated with respect to various time points for end of study, different treatment effects and different values for percentage of switching. In each scenario, survival times for 500 patients were generated and 1000 iterations were run for each scenario. Table 1 shows the summary of characteristics of the scenarios in this simulation study.

**Table 1:** Specifications of various scenarios in this simulation study.

Variables	Number of levels	Description
Entrance time	1	Uniformly from zero to 20 months
Switching time	1	Generated right after observing a disease progression
True treatment effect for the event of interest	1	2 (equivalent to HR = 0.5)
True treatment effect for the competing event	3	0.5, 1 and 1.5
Sample size	1	250 subjects per group
Switching percent	3	30%, 60% and 90%
End of study time (months)	2	30 and 90 (months)
Number of replications	1	1000

To justify the sample size, the simulation was repeated for some scenarios with a sample size of 1000 patients. Similar results were observed, but not reported here.

### Performance measures

Data for 500 subjects were generated 1000 times as specified in Table 1. Percentage of censoring, percentage of coverage (the number of times that the calculated confidence interval contains the true value of HR, divided by the number of runs), HR (average HR from different replications), empirical standard deviation of HRs (SD), mean of square errors $\left( {\frac{{\sum \left( {\hat{\theta } - mean\left( {\hat{\theta }} \right)} \right)^{2} }}{\# Run}} \right)$ and the percent of relative bias $\left( {\frac{{\left| {\hat{\theta } - \theta } \right|}}{\theta } \times 100} \right)$ were calculated ($\widehat{\theta } \; is \; estimator \; and \; \theta \; is \; parameter)$^32^.

## Simulation study results

In this section, the details of 18 scenarios in three tables were presented based on different treatment effects for the competing event with respect to the event of interest $(exp({-\psi }_{E})=2$ equivalent to ${HR}_{E}=0.5$).

The name of each method was abbreviated as follows: ITT for Intention to treat, PP-cen for Censor at switch, RPSFT for Rank preserving structural failure time model and “com” for the composite endpoint.

### Scenarios with ${\varvec{e}}{\varvec{x}}{\varvec{p}}({-{\varvec{\psi}}}_{{\varvec{C}}})=1$ equivalent to ${{\varvec{H}}{\varvec{R}}}_{{\varvec{C}}}=1$

Table 2 shows details of scenarios 1 to 6, where hazard rates are identical between treatment groups for patients with competing event. Generally, methods based on CSH estimate the HR more precise than methods based on composite endpoint. The RPSFT method estimates the HR with the minimum bias in all scenarios. Moreover, the shorter the study period or the higher percentage of switching, the larger bias is observed. The ITT method has less bias compared to the PP-cen method. By raising the percentage of switching in PP-cen method, the bias of estimates for HR is considerably increased, which shows occasional completely wrong estimates. Additionally, the Percentage of coverage for the PP-cen and ITT are reduced, when the percentage of switching is increased.

**Table 2:** Results of Scenario 1 to 6 with ${HR}_{E}=0.5$ and ${HR}_{C}=$ *1.*

Switching percent (%)	End of study time	Method	HR_E_*	Percent of censored patient	Coverage percent	SD	MSE	Relative bias (%)
30	90	RPSFT	0.56	0.58	0.80	0.082	0.010	12.06
		ITT	0.58	0.49	0.78	0.068	0.011	16.01
		PP-cen	0.63	0.57	0.63	0.088	0.025	25.99
		RPSFT-com	0.63	0.35	0.53	0.083	0.023	25.50
		ITT-com	0.67	0.28	0.23	0.071	0.035	34.57
	90	PPcen-com	0.77	0.39	0.05	0.089	0.080	53.63
		RPSFT	0.54	0.33	0.84	0.076	0.007	8.06
		ITT	0.56	0.30	0.82	0.061	0.008	12.86
		PP-cen	0.61	0.41	0.67	0.071	0.016	21.19
		RPSFT-com	0.64	0.01	0.30	0.068	0.024	27.51
		ITT-com	0.69	0.01	0.06	0.063	0.040	37.89
60	30	PPcen-com	0.74	0.16	0.03	0.074	0.063	47.90
		RPSFT	0.57	0.57	0.77	0.110	0.017	13.73
		ITT	0.62	0.51	0.65	0.078	0.020	23.76
		PP-cen	0.95	0.67	0.02	0.153	0.228	90.47
		RPSFT-com	0.65	0.35	0.42	0.101	0.034	30.75
		ITT-com	0.74	0.30	0.06	0.078	0.063	47.69
	90	PPcen-com	1.16	0.51	0.00	0.159	0.464	132.53
		RPSFT	0.55	0.33	0.79	0.090	0.010	9.55
		ITT	0.62	0.30	0.53	0.067	0.018	23.49
		PP-cen	0.86	0.53	0.04	0.123	0.143	71.64
		RPSFT-com	0.66	0.01	0.24	0.085	0.033	32.24
		ITT-com	0.75	0.01	0.01	0.070	0.069	50.84
90	30	PPcen-com	1.05	0.31	0.00	0.126	0.315	109.34
		RPSFT	0.59	0.57	0.69	0.131	0.025	17.46
		ITT	0.68	0.52	0.37	0.091	0.042	36.78
		PP-cen	3.38	0.76	0.00	1.090	9.471	575.56
		RPSFT-com	0.69	0.35	0.33	0.130	0.054	38.6
		ITT-com	0.81	0.31	0.01	0.087	0.101	61.31
	90	PPcen-com	4.06	0.63	0.00	1.003	13.65	711.12
		RPSFT	0.56	0.33	0.70	0.107	0.015	11.9
		ITT	0.68	0.30	0.21	0.073	0.036	35.13
		PP-cen	2.78	0.65	0.00	0.769	5.795	456.23
		RPSFT-com	0.71	0.01	0.15	0.112	0.055	41.3
		ITT-com	0.83	0.01	0.00	0.074	0.112	65.24
		PPcen-com	3.37	0.46	0.00	0.764	8.844	574.81

*Cause-specific hazard ratio.

According to the results in Table 2, the standard deviation (SD) of estimates in ITT method is slightly less than the SD in RPSFT method. When comparing the SD of estimates for different methods and scenarios, some methods may show the smallest value for SD, but only the RPSFT estimates have the lowest MSE among all methods and for all scenarios. Moreover, the percentage of coverage for RPSFT method has the highest value. Besides, the percentage of censored patients for methods based on CSH is generally higher than the corresponding percent in methods based on composite endpoints.

### Scenarios with ${\varvec{e}}{\varvec{x}}{\varvec{p}}(-{{\varvec{\psi}}}_{{\varvec{C}}})=0.5$ is equivalent to ${{\varvec{H}}{\varvec{R}}}_{{\varvec{C}}}=2$

Table 3 shows details of scenarios 7 to 12, in which the treatment effect for patients with competing event is the inverse of the treatment effect for patients with the event of interest; i.e. the experimental treatment for patients with competing event is harmful. That is, the hazard rate in experimental treatment is twice as big as in the standard treatment. The proposed methods based on CSH performed well for all scenarios, among which the RPSFT method was the best. The HR for RPSFT method had the least bias and the MSE. In addition to that, the coverage percentage for this method is the highest among all cases.

**Table 3:** Results of Scenario 7 to 12 with HR_C_ = 2.

Switching percent (%)	End of study time	Method	HR_E_*	Percent of censored patient	Coverage percent	SD	MSE	Relative bias
30	30	RPSFT	0.54	0.57	0.90	0.085	0.009	7.47
		ITT	0.55	0.47	0.89	0.07	0.008	10.47
		PP-cen	0.63	0.56	0.63	0.088	0.025	26.09
		RPSFT-com	0.72	0.35	0.18	0.097	0.057	43.49
		ITT-com	0.75	0.30	0.05	0.084	0.071	50.56
		PPcen-com	0.87	0.40	0.01	0.106	0.145	73.3
	90	RPSFT	0.52	0.30	0.91	0.067	0.005	4.24
		ITT	0.57	0.25	0.78	0.06	0.008	13.05
		PP-cen	0.60	0.38	0.64	0.071	0.016	20.9
		RPSFT-com	0.74	0.01	0.04	0.080	0.062	47.26
		ITT-com	0.78	0.01	0.00	0.070	0.083	55.77
		PPcen-com	0.83	0.16	0.00	0.082	0.118	66.66
60	30	RPSFT	0.55	0.56	0.83	0.097	0.012	10.93
		ITT	0.62	0.49	0.63	0.079	0.020	23.87
		PP-cen	0.97	0.66	0.01	0.158	0.248	94.39
		RPSFT-com	0.77	0.35	0.12	0.121	0.090	54.79
		ITT-com	0.83	0.32	0.00	0.090	0.118	66.42
		PPcen-com	1.34	0.52	0.00	0.190	0.734	167.01
	90	RPSFT	0.53	0.30	0.84	0.080	0.007	5.29
		ITT	0.63	0.25	0.45	0.064	0.02	25.04
		PP-cen	0.86	0.51	0.02	0.112	0.143	72.24
		RPSFT-com	0.80	0.01	0.01	0.107	0.10	59.5
		ITT-com	0.86	0.01	0.00	0.077	0.135	71.81
		PPcen-com	1.19	0.31	0.00	0.133	0.495	138.1
90	30	RPSFT	0.58	0.56	0.73	0.119	0.021	16.46
		ITT	0.69	0.50	0.34	0.089	0.042	37.16
		PP-cen	3.44	0.76	0.00	0.974	9.586	587.79
		RPSFT-com	0.85	0.36	0.08	0.168	0.151	70.02
		ITT-com	0.91	0.33	0.00	0.099	0.175	81.31
		PPcen-com	4.69	0.64	0.00	1.227	19.10	838.91
	90	RPSFT	0.54	0.30	0.72	0.118	0.016	8.92
		ITT	0.69	0.25	0.16	0.072	0.04	37.4
		PP-cen	2.86	0.64	0.00	0.739	6.098	471.26
		RPSFT-com	0.90	0.02	0.01	0.162	0.187	80.26
		ITT-com	0.94	0.01	0.00	0.085	0.202	88.26
		PPcen-com	3.90	0.46	0.00	0.875	12.35	680.54

*Cause-specific hazard ratio.

As the percentage of switching increases, the bias of estimates in PP-cen method not only increases considerably, but also estimates the HR in the opposite direction especially in scenario with 90% switching. Also the coverage percentage is significantly reduced for PP-cen in particular.

### Scenarios with ${\varvec{e}}{\varvec{x}}{\varvec{p}}({-{\varvec{\psi}}}_{{\varvec{C}}})=1.5$ is equivalent to HR_C_ =  0.6

Table 4 shows details of scenarios 13–18, in which the treatment effect for patients with competing event (${e}^{{-\psi }_{C}}=1.5$) is less than for the patients with the event of interest (${e}^{{\psi }_{E}}=2$). The hazard rate in the experimental treatment is 1.5 times as big as the standard treatment, i.e. it can be said that the treatment effect is similar for both the event of interest and the competing event. Similar to the previous scenarios, the methods based on CSH, especially the RPSFT method, performed well compared to composite endpoints. In all scenarios, the relative bias and the MSE for RPSFT were the lowest. The coverage percentage of RPSFT in all scenarios was higher than the coverage percentage for all other methods.

**Table 4:** Results of Scenario 13 to 18 with HR_C_= 0.6

Switching percent (%)	End of study time	Method	HR_E_*	Percent of censored patient	Coverage percent	SD	MSE	Relative bias
30	30	RPSFT	0.54	0.59	0.88	0.087	0.009	8.35
		ITT	0.56	0.51	0.85	0.074	0.010	12.86
		PP-cen	0.63	0.59	0.64	0.091	0.025	25.69
		RPSFT-com	0.56	0.35	0.80	0.074	0.010	12.85
		ITT-com	0.61	0.27	0.53	0.065	0.017	22.7
		PPcen.com	0.69	0.38	0.23	0.079	0.041	37.39
	90	RPSFT	0.54	0.36	0.85	0.078	0.007	7.29
		ITT	0.57	0.34	0.80	0.066	0.009	13.34
		PP-cen	0.60	0.44	0.72	0.077	0.016	20.04
		RPSFT-com	0.56	0.01	0.75	0.061	0.008	12.4
		ITT-com	0.62	0.01	0.37	0.058	0.018	24.25
		PPcen-com	0.66	0.16	0.26	0.067	0.029	31.37
60	30	RPSFT	0.57	0.58	0.67	0.092	0.012	14.44
		ITT	0.63	0.52	0.59	0.085	0.024	26.23
		PPcen	0.94	0.68	0.04	0.159	0.223	88.99
		RPSFT-com	0.59	0.35	0.67	0.091	0.016	17.57
		ITT-com	0.68	0.29	0.2	0.074	0.039	36.44
		PPcen-com	1.03	0.51	0.00	0.140	0.303	106.43
	90	RPSFT	0.58	0.35	0.66	0.091	0.010	16.47
		ITT	0.64	0.34	0.44	0.071	0.024	27.31
		PP-cen	0.86	0.54	0.04	0.125	0.146	72.31
		RPSFT-com	0.59	0.01	0.54	0.075	0.014	18.18
		ITT-com	0.70	0.01	0.06	0.065	0.044	39.75
		PPcen-com	0.94	0.31	0.00	0.112	0.209	88.58
90	30	RPSFT	0.61	0.58	0.6	0.149	0.033	21.07
		ITT	0.70	0.54	0.31	0.092	0.048	40.03
		PP-cen	3.33	0.76	0.00	1.128	9.279	565.91
		RPSFT-com	0.62	0.35	0.56	0.115	0.027	23.24
		ITT-com	0.75	0.30	0.04	0.082	0.071	50.8
		PPcen-com	3.59	0.63	0.00	0.963	10.49	618.44
	90	RPSFT	0.62	0.35	0.57	0.146	0.035	23.02
		ITT	0.70	0.34	0.17	0.079	0.045	39.49
		PP-cen	2.83	0.65	0.00	0.844	6.15	466.34
		RPSFT-com	0.62	0.01	0.44	0.096	0.024	24.19
		ITT-com	0.77	0.01	0.00	0.070	0.076	53.32
		PPcen-com	3.09	0.45	0.00	0.718	7.222	517.97

*Cause-specific hazard ratio.

## Case study: a randomized clinical trial in colorectal cancer

Here, this proposed methodology is illustrated using data on the panitumumab colorectal cancer clinical trial^33^. This clinical trial compares the efficacy and safety of panitumumab plus best supportive care versus best supportive care alone in colorectal cancer patients. Patients were randomly assigned either to the treatment or the control group. The treatment group received panitumumab treatment at randomization time, whilst the control group received panitumumab at any time and who had disease progression. Among the 223 patients on the control group, 201 patients had disease progression, of which 167 switched over to the treatment group. Also, among 231 patients on the treatment group, 186 patients had disease progression. The switching rate for the best supportive care alone group was 167/223 = 75%.

The endpoint considered here was the time from entry to the study until death, following disease progression or death without disease progression. Due to noncompliance in this patient’s population, this study developed new method for estimating the treatment effect in the presence of a competing risk.

Due to lack of access to this trial, a Monte Carlo simulation study was conducted to generate data set. The median time to disease progression, time to death and time to death for switching patients was 53, 190 and 49 days, respectively. The survival time was generated for these endpoints from the exponential distribution with parameters log(2)/53, log(2)/190 and log(2)/49, respectively. The results showed that for the proposed method, the HRs for death following disease progression and death without disease progression were ${h}_{m1}/{h}_{s1}=0.22$ and ${h}_{m2}/{h}_{s2}=0.96$; and the HRs for death in composite endpoints was ${h}_{m}/{h}_{s}=0.29$. Also, in the ITT approach, the HRs ${h}_{m1}/{h}_{s1}=0.4$, ${h}_{m2}/{h}_{s2}=0.74$ and ${h}_{m}/{h}_{s}=0.47$ were obtained for death following disease progression, death without disease progression and death (composite endpoints) respectively. Furthermore, When using PP-cen approach,${h}_{m1}/{h}_{s1}=0.9$, ${h}_{m2}/{h}_{s2}=0.44$ and ${h}_{m}/{h}_{s}=0.99$ were estimated for those endpoints. Greater emphasis was placed on the results of the proposed approach because not only it takes noncompliance but also CR into account.

## Discussion

In some RCTs, patients are allowed to switch from the standard to the experimental treatment. Since, in the presence of treatment switching, the estimate of the treatment effect might be biased, an appropriate method for adjusting for noncompliance has to be chosen. The advanced analysis such as RPSFT method is often performed. But so far all investigated methods (such as ITT, PP and RPSFT) have been used in classical time-to-event data when there is only one event type. In the presence of multiple events, CR techniques should be applied in order to obtain reliable estimates. So, the proposed method here can be considered as an extension of the RPSFT method and GE algorithm when there is a competing event.

In this study, to assess the association between treatment groups and the event of interest, a CSH model was used. It is assumed that the proportional CSH and hence, the proportional SDH model are mis-specified. The motivation for this has been twofold: first, modeling proportional CSHs has been the standard approach in CR analysis. Second, the CSHs completely determines the stochastic manner of the CR process^24,25^. In all simulations, the RPSFT, ITT and PP methods were also applied to composite endpoints. Their results were compared to the results of methods based on CSH in the presence of CR.

The simulation results showed that in all scenarios, the estimates of treatment effect in CSH methods were quite better than the estimates from methods based on composite endpoints. Also our simulation study showed that in all scenarios, the estimation of the treatment effect in RPSFT has a better performance compared to the other methods in the presence of CR.

In all scenarios, the percentage of censored patients in methods based on CSH was higher than the percentage in other methods with composite endpoint. Moreover, in all scenarios, the percentage of censoring patients in ITT is less than RPSFT and PP.cen. This makes sense, because ITT method only censors survival time of patients who have administrative censoring. But in RPSFT method, in the process of re-censoring, some people who were not previously censored are censored after applying this procedure. In PP.cen, in addition to patients who had administrative censoring, the survival of those patients who switched over study period had to be censored too. Hence, the censoring rate in this method was high in all scenarios.

In all investigated scenarios, the coverage probability in CSH methods was higher than coverage probability from methods based on composite endpoints and the coverage probability of RPSFT was higher than other methods.

The results showed that the higher the percentage of switching was, the larger the bias of estimates in all methods was. This simulation study also revealed that in all scenarios, the RPSFT had less bias compared to the other methods.

Other simulation studies, such as the present study, recommended the use of the RPSFT for estimating of the treatment effect in the presence of noncompliance^3^. In Scenarios 1 to 6 with $exp(-{\psi }_{C})=1$, which was the null case for the competing event, the RPSFT compared to ITT and PP was adequately better. These scenarios were similar to some trials, in which the treatment had no effect on the competing disease process. In scenarios with low percentage of switching, the ITT estimates were close to RPSFT estimates. Thus, ITT can be a good alternative for RPSFT. In scenarios 7 to 12 with ${exp(-\psi }_{C})=0.5$ showed that the experimental treatment for patients with competing event was harmful. The RPSFT acted optimally compared to other scenarios such as the ITT and PP methods. In a real RCTs, these scenarios are more factual, since the purpose of the trial is to evaluate the effect of treatment on the event of interest, not on the competing event. In Scenarios 13 to 18 with $exp({-\psi }_{C})=1.5$, the results of CSH methods were close to the results of methods based on composite endpoints, because, the effect of experimental treatment on the event of interest was almost similar to the effect on competing event. So, survivals of all patients were getting close and the censoring in CSH approach does not improve the results compared to methods based on composite endpoints.

In case study, the results showed that the HR in proposed RPSFT was less than ITT and PP.cen. Also, the HR in all methods based on CSH were less than HR based on composite endpoint. The reason for this can be explained by the fact that the effect of immediate panitumumab treatment on patients’ survival is positive and the hazard in this group was less than control group. So in the delayed group with noncompliance and receiving combined treatment, HR has been unrealistically decreased and survival has been increased.

For simplicity, it was assumed that the patients could only switch from the standard treatment to the experimental treatment. It was also assumed that each patient could switch only once during the study period. The administrative censoring was assumed to be the only source of censoring.

The main limitation of this study was the use of “common treatment effect” as a key assumption of the RPSFT method. Also the other limitation of this study was inaccessibility to a real clinical trial dataset, to assess the performance of the estimation methods of treatment effect.

The programing codes written for the simulation study are available from the corresponding author, upon request.

The results of this simulation study showed that in RCTs with noncompliance, the RPSFT method based on CSH was a mighty method for estimating the treatment effect in the presence of CR in all scenarios. More research work regarding the extension of RPSFT based on SDH should be undertaken in the future. The present proposed method can be applied for non-administrative censoring or informative censoring as well as competing risks. All statistical analysis and the two-dimensional GE algorithm were programed in R.3.5.3.

## Data Availability

The datasets generated during and/or analyzed during the current study are available from the corresponding author on reasonable request but restrictions apply to the availability of these data, which were used under license for the current study, and so are not publicly available. Data are however available from the authors upon reasonable request and with permission of the corresponding author. Also the data in Case Study was used from results of phase III mCRC trial comparing panitumumab monotherapy to best supportive care (Trial registration: ClinicalTrials.gov NCT00113763 NCT00113776) (33).

## Code availability

The codes that support the findings of this study are available from Ghodratollah roshanaei (Email: gh.roshanaei@umsha.ac.ir) but restrictions apply to the availability of these codes, which were used under license for the current study, and so are not publicly available. Codes are, however available from the authors upon reasonable request and with permission of the corresponding author.
